# MEDICAID FINANCING IN ASSISTED LIVING AND CHARACTERISTICS OF MEDICARE DUAL-ELIGIBLE RESIDENTS

**DOI:** 10.1093/geroni/igz038.1102

**Published:** 2019-11-08

**Authors:** Chanee D Fabius, Portia Y Cornell, Kali S Thomas

**Affiliations:** 1 Johns Hopkins University, Baltimore, Maryland, United States; 2 Brown University, Providence, Rhode Island, United States; 3 Providence VA Medical Center, Providence, Rhode Island, United States

## Abstract

An increasing number of assisted living (AL) residents rely on Medicaid waivers or state plans to pay for personal care and other supportive services. States may finance services for duals residing in AL through Medicaid waivers and state plans, but the availability of coverage varies – some states offer little to no Medicaid coverage for services in AL, and others offer multiple pathways to receiving assistance. Little is known about duals in AL, including how many have access to AL and the quality of care they receive there. The present study compares the characteristics of Medicare beneficiaries residing in large AL settings, by dual-eligibility status, and investigates the variability in the share of duals in AL among states. We identified 586,397 Medicare beneficiaries residing in AL in 2014. Medicare claims were used to measure health characteristics and health care utilization. Duals represented 16% of AL residents in our cohort. Compared to non-duals, duals were more often older adults of color (24 vs 4%), and more likely to qualify for Medicare due to disability (46 vs 7%). Duals had higher rates of hospitalizations (24 vs 21%) and skilled nursing facility use while in AL (11 vs 10%), and more chronic conditions than non-duals. States varied in the share of AL residents who are duals, ranging from 6% in New Hampshire to 41% in New York. State policies that may contribute to variation in the prevalence of duals in AL and implications of these findings for policy-makers and residents will be discussed.

